# How Do Gender and Marital Status Influence Village Savings and Loan Associations Related Gains and Usage? A Cross‐Sectional Study in Fort Portal, Uganda

**DOI:** 10.1002/hsr2.70597

**Published:** 2025-03-27

**Authors:** HaEun Lee, Alex Shen, Alia Dada, Isabel Gilbertson, Cheryl A. Moyer, Donah Asiimire

**Affiliations:** ^1^ Department of Systems, Populations and Leadership University of Michigan School of Nursing Ann Arbor Michigan USA; ^2^ Department of Statistics and Data Science Carnegie Mellon University Dietrich College of Humanities and Social Sciences Pittsburgh Pennsylvania USA; ^3^ Global Health Epidemiology University of Michigan School of Public Health Ann Arbor Michigan USA; ^4^ Learning Health Sciences Obstetrics and Gynecology University of Michigan Ann Arbor Michigan USA; ^5^ Department of Economics, Statistics, and Tourism Management Bishop Stuart University Mbarara Uganda

**Keywords:** gender, marital status, microfinance, savings group, sub–Saharan Africa, usage, village savings and loans association

## Abstract

**Background and Aims:**

Village savings and loan association (VSLA) is a widely adopted informal microfinance mechanism designed to empower women financially. However, limited studies have examined how gender and women's marital status influence VSLA‐related gains and fund usage. This study aims to assess whether rural Ugandan VSLA members' gender and marital status impact their financial gains and fund usage.

**Methods:**

A cross‐sectional survey was conducted among 132 participants in five VSLAs in Fort Portal, Uganda. Participants were categorized as married women, single/never married women, widowed/separated women, and VSLA‐participating men. The survey included questions on asset ownership, expenditures, and usage of VSLA funds for income‐generating activities (IGAs) and life events (LEs). Descriptive analysis and logistic regression were used to assess relationships between gender, marital status, asset ownership, and fund usage.

**Results:**

VSLA‐participating women owned fewer overall assets (AOR: 0.81; 95% CI: 0.71–0.92) than men but utilized VSLA funds more extensively (AOR: 1.40; 95% CI: 1.16–1.70), particularly for LEs (AOR: 1.61; 95% CI: 1.29–2.01). Single/never married women owned fewer assets (AOR: 0.83; 95% CI: 0.70–0.98) compared to married women. Their asset ownership leaned toward LEs (AOR: 0.73; 95% CI: 0.53–0.99), while VSLA fund usage prioritized IGAs (AOR: 1.84; 95% CI: 1.00–3.40) compared to married women.

**Conclusion:**

Significant differences exist in VSLA fund usage and gains based on gender and marital status. Women, particularly single women, face asset ownership disparities but strategically utilize VSLA funds. Future studies should investigate gender dynamics within VSLAs and explore tailored strategies to address the specific needs of women across marital statuses for inclusive financial empowerment.

## 1 Introduction

Currently, 700 million people live in extreme poverty, defined as living on less than $2.15 a day [1]. Just over half of these people live in sub‐Saharan Africa [1]. Village savings and loan association (VSLA) is an informal microfinance mode that is aimed to deliver financial services such as savings and loans to people living in rural areas with no or limited access to formal financial services [2]. It is also shown to be effective strategy to strengthen financial and climate resilience, particularly in mitigating food insecurity among vulnerable population [3]. VSLA shares similar characteristics with other savings and credit groups (SCGs) such as Savings and Internal Lending Communities (SILCs) [4], self‐help groups (SHGs) [5], and Savings Group (SGs) [6], in that it is low risk, self‐managed, and self‐financed [6, 7].

VSLA often consists of 15–30 or more group members who meet regularly (weekly, bi‐weekly, or monthly) with the objective to bring money to the group to save the amount determined by the group. With the accumulated savings, members can choose to take out loans for various reasons. The two main reasons for loans are: (1) to deal with life events (LEs) and (2) to invest in income generative activities (IGAs) [7]. Some examples of LE‐related loans, also known as social loan or emergency loan, include paying for children's school fees, purchasing clothing and food, accessing medical services, and dealing with a family member's death. IGA‐related loans include purchasing land, seeds, farming equipment, livestock, and business‐based resources. The ultimate goal of IGAs is to generate more income for the individual and the group members, allowing the individual to gain financial independence and invest money back into the VSLA group.

VSLA and other SCGs often target women as a key demographic group due to women and girls bearing the burden of poverty due to gender roles and social exclusion, which often limits their ability to reach their full potential [2, 8, 9]. Research has found that when women gain financial resources and have the economic decision‐making power, they and their families benefit through improved familial nutrition, hygiene, children's education, and health outcomes [8]. Furthermore, women's access to loans leads to the ability for them to participate in economic activities such as raising and selling animals, cooking and selling food, starting small businesses, and ultimately allowing them to generate sustainable income for themselves and their families [10].

While the literature generally suggests that women's participation in VSLA leads to gender equality and female empowerment, there are contrasting findings. Women's gain of financial resources can also lead to their exploitation. When women have access to loans and savings but do not have control over their resources, they are at risk of being financially exploited [8, 11, 12]. Moreover, men may feel as if their masculinity is being threatened by women's increased financial resources, which can lead to an increased risk of domestic violence [13]. Hence, when gender is examined within the context of VSLAs and other SCGs, it is assessed in relation to their husbands rather than other men participating in VSLA. A systematic review examining the impact of SHG participation on women's empowerment found that when attempting to measure women's economic empowerment, questionnaires focus on assessing women's decision‐making power in relation to that of their husbands [14]. While it is important to understand how decisions are made within households, this method assumes that all women are married and fails to accurately capture single, separated, or widowed women's economic empowerment.

In fact, in the majority of the SCGs interventions, women are treated as a homogenous group, as married women with children. However, the single, separated, or widowed women are more marginalized, and their marital status significantly influence their VSLA related activities. In sub‐Saharan African countries, women's marital status carries profound socioeconomic and cultural significance. In Uganda, for instance, land is a critical asset in rural areas, and many women gain access to it—or claim some level of ownership—through marriage [15]. Land inheritance practices typically prioritize sons over daughters, leaving most women without direct access to inherited land [15]. Widows face additional barriers, as they can only claim land that belonged to their late husbands, often relying on having a son to eventually inherit and secure the land upon reaching adulthood. As a result, asset rights overwhelmingly favor married women, disadvantaging single women and widows [15].

Given these dynamics, we hypothesize that women's marital status significantly influences access to and use of VSLA loans. However, research examining the implications of women's marital status remains limited. Even though several SCGs target widows or consider marital status, few studies have explored how these dynamics impact women's participation, SCG‐related outcomes, or how programs can be tailored to better support women across different marital statuses. For example, a study conducted by Sooryamoorthy (2005) in Kerala, India found that married women had a higher amount of average money saved each week, a higher total amount of money taken out as loans, and a higher total amount of savings compared to single women [16]. Moreover, findings from Copestake et al (2001) demonstrate that compared to single women, married women in Zambia had 26% higher profit from businesses started with SCG related resources [17].

While these studies found that marital status indeed plays a significant role in various SCGs participation and outcomes, they did not further examine the different nuances among single women, whether they have never been married or are separated or widowed nor the nuances among married women. Therefore, the purpose of this study was to examine how gender (men vs. women) and marital status (married, single, separated/widowed) influence wealth and VSLA related financial expenditures.

## 2 Conceptual Models

The Theory of Intersectionality [18] and Social Capital Theory [19, 20] provide useful frameworks for understanding how VSLA participation is shaped by the overlapping influences of gender, marital status, and financial access. Intersectionality recognizes that individuals experience multiple, interlocking systems of privilege and disadvantage based on their identities, such as gender and marital status [18, 21]. In the context of this study, women's financial agency and economic opportunities are not shaped solely by gender but also by their marital status, which influences their access to financial resources, decision‐making power, and economic security. Social Capital Theory asserts that individuals derive benefits from their membership in social networks, such as VSLAs, which serve as financial and support systems [19, 20]. However, access to and the benefits derived from social capital are not equally distributed; they are influenced by intersecting identities and social norms. For example, married, single, and widowed women may experience different levels of economic autonomy and access to VSLA funds based on societal expectations and the financial structures available to them. Together, these theories help frame VSLAs not just as financial institutions but as socially embedded systems where the intersection of gender and marital status influences who benefits, how they benefit, and what constraints they face in achieving financial empowerment.

## 3 Methods

A cross‐sectional survey was collected from randomly selected participants from five VSLA groups in Fort Portal, Uganda. The new VSLA cycle was underway (since Jan 2023) and the VSLA surveys were collected at approximately halfway through the VSLA cycle in June 2023. Thirty‐five women from each marital status (married, single/never married, and widowed/separated) and VSLA participating men were recruited for the survey. The surveys were implemented by Ugandan research assistants (RAs) who received a 1‐day intensive training.

### 3.1 Setting

Fort Portal is in Kabarole district in Western Uganda, with a population of approximately 54,000 people [22]. Fort Portal is a crucial market and processing center for cotton, peanuts, corn, coffee, and tobacco [23]. Industrial products include furniture, soap, shoes, metal goods, and textiles. Women are heavily involved in producing these products and often join VSLAs to purchase materials for handcrafts and agricultural products [22]. The research team has an established relationship with the five VSLAs in Fort Portal, Uganda. Site visits and information related to each VSLA were collected before study implementation in September 2022.

### 3.2 Sampling

Five VSLAs were included in this study. Group sizes varied among the VSLAs, ranging from 46 to 119 participants. We developed a roster with the accumulated VSLA participants with the help from each VSLA's management committee members. From the accumulated roster, we stratified the participants based on their marital status and randomly selected 35 participants per marital status using an online random number generator. Additionally, we recruited 35 VSLA participating men. Once the participants were selected, the VSLA management committee members recruited the individuals via phone.

### 3.3 Tool

The survey consisted of three sections. The first section determined participant eligibility, and the second section contained demographic questionnaires including marital status, head of household, age, education level, religion, reproductive history, and so forth. The third section inquired about household wealth and expenditures, such as ownership of various household assets, type of housing materials and water supply, ability to pay children's school fee, and healthcare service fees. Following each of the questions, we also asked whether money from VSLA was used to purchase/improve/access each of the items. Part of the third section, regarding asset ownership, was adapted from the Demographic and Health Survey (DHS), commonly used as means to estimate household wealth in low‐ and middle‐income countries [24, 25]. Following each of the asset ownership questions, the survey asked, “Did you use the VSLA money to purchase this item/pay for this fee?” as a follow‐up to capture VSLA related information. Additionally, researchers added questions regarding healthcare expenses.

### 3.4 Data Collection

Each VSLA survey took approximately 60 min to complete. The RAs traveled to a location where the VSLA group usually meets. First, informed consent was read and collected on paper. If the participant was not able to write their own name, their fingerprints were collected to serve as consent. A hardcopy of the consent form was given to the participant. If the participant had enough literacy to complete the survey, they answered the questions in a quiet space. If the participant had low literacy, the RAs read each question and associated answer options to the participant. All VSLA surveys were collected on paper then transferred to Excel sheets.

### 3.5 Measures

The primary outcomes of interest are: (1) overall assets, (2) IGA assets, (3) LEs assets, (4) overall usage, (5) IGA usage, and (6) LE usage. Using the survey questionnaires, we developed the six dependent variables. Overall assets is the fraction of assets/expenditures by the survey participants over the total number of assets/expenditures listed in the survey. From the overall assets variable, we further developed IGA assets and LE assets. IGA assets is a fraction of the IGA related assets/expenditures over the total IGA related assets/expenditure questions. IGA assets included questions such as ownership of land, farming equipment, and livestock. LE assets is a fraction of the LE related assets/expenditures over the total LE related assets/expenditure questions. These include children's school fees, hospital fees, and house improvement projects. In total, IGA assets consist of 23 questions from the survey and LE assets consist of 34 questions.

Similarly, overall usage is the usage of VSLA funds to purchase/pay for any assets/expenditures included in the overall assets. It is the number of asset/expenditure items paid using VSLA funds over the total number of assets/expenditures marked as owned by the participant, which means that assets that were not owned in the first place are not included in the overall usage calculation. From overall usage we further developed IGA usage and LE usage, examining which category the VSLA funds were used. While many of the questions in asset and usage variables overlap, additional questions such as categorized health expenditures related to pregnancy and childbirth were asked if applicable to the participant. In total, IGA usage consisted of 25 possible questions and LE usage consisted of 46 possible questions, depending on each participant.

### 3.6 Analysis

We were interested in quantifying the relationship between each outcome of interest above and other variables collected in the survey, specifically those related to the participant's marital status. First, we used means, standard deviations, counts, and percentages to do exploratory analysis of the demographic characteristics of the participants. Of particular interest is any marginal association between marital status and the outcomes of interest, which provide context and foundation for more principled analysis between these two variables.

Marginal correlations, of course, are subject to confounding effects. For example, participant age may be a causal predecessor of marital status, and simultaneously a causal predecessor of VLSA participation. In this case, any observed correlation between marital status and VSLA participation cannot be interpreted as causal. To address this issue, we use modeling techniques that allow us to control for potential confounders. We use logistic regression as our statistical model due to its interpretability and our relatively small sample size.

We first use the logistic regression model below to examine the relationship between the six outcomes of interest and participant gender, controlling for potential confounders:

$$\mathrm{logit}\left(\text{Pr}\left[Y_{i}=1\right]\right)=\beta_{0}+\beta_{1}\mathrm{Femal}e_{i}+\gamma {\;X}_{i}$$

The model above is fit for each of the six outcomes of interest. For example, in the model for overall asset ownership, $\text{Pr}\left[Y_{i}=1\right]$ is the probability that respondent *i* owns any asset listed in the survey. As is convention with logistic regression, we take the *logit* (e.g. the log‐odds or inverse logistic function) of the left side so that effects of interest can be interpreted as odds ratio On the right side, $F\mathrm{emal}e_{i}$ is a dummy variable to indicate whether the respondent is female. $X_{i}$ is a collection of control variables for a respondent (*i)*. Specifically, we include a respondent's VSLA group, age, household size, religious background, and education level. $\beta_{0}$ is the intercept term, $\beta_{1}$ is the coefficient of interest, and γ are the associated coefficients for the control variables. Specifically, our estimate of $\beta_{1}$ indicates the adjusted odds ratio (AOR) for participant gender on each outcome of interest, controlling for the potential confounders listed above.

Next, we use the model below to examine the association between women's marital status and the six dependent variables:

$$\mathrm{logit}\left(\text{Pr}\left[Y_{i}=1\right]\right)=\beta_{0}+\beta \;{\mathrm{MS}}_{i}+\gamma \;X_{i}$$

${\mathrm{MS}}_{i}$ represents the respondent's marital statuses for women or as a man. β are the associated coefficients for each level of this categorical variable (married, widowed/divorced, single/never married, and male). As with the previous model, we fit this model for each of the six dependent variables on the left side of the equation. Our estimate of the coefficients of interest β will likewise indicate the AOR participant marital status on outcomes of interest (relative to the baseline category), controlling for the potential confounders.

Finally, we examine how the relative IGA vs LE asset ownership and VLSA usage is influenced by both gender and marital status. To do so, we fit the following two logistic regression models.

$$\mathrm{logit}\left(\text{Pr}\left[{\mathrm{IGA}}_{i}=1\right]\right)-\mathrm{logit}\left(\text{Pr}\left[{\mathrm{LE}}_{i}=1\right]\right)=\beta_{0}+\beta_{1}\mathrm{Femal}e_{i}+\gamma {\;X}_{i}$$

$$\mathrm{logit}\left(\text{Pr}\left[{\mathrm{IGA}}_{i}=1\right]\right)-\mathrm{logit}\left(\text{Pr}\left[{\mathrm{LE}}_{i}=1\right]\right)=\beta_{0}+\beta \;{\mathrm{MS}}_{i}+\gamma \;X_{i}$$

For each model above, we estimate parameters for two sets of dependent variables. The first set of dependent variables is IGA asset ownership and LE asset ownership. In this case, the model dependent variables represent the difference between IGA asset ownership and LE asset ownership. Specifically, $\text{Pr}\left[{\mathrm{IGA}}_{i}=1\right]$ is the probability that respondent *i* owns an IGA asset, and $\text{Pr}\left[{\mathrm{LE}}_{i}=1\right]$ is the probability that respondent *i* owns a LE asset. Other variables and coefficients retain their same definition from the previous models. After computing these two model fits, we move onto the second set of dependent variables: VLSA usage for IGA assets and VSLA usage for LE assets. Each model is then fit again with this pair of dependent variables, resulting in a total of 4 model fits for this last pair of models.

The structure of these last two models was chosen to give the parameters of interest a suitable interpretation. Instead of AORs for each of the six outcomes of interest, the coefficients of interest are “Adjusted Category Odds Ratio Factors” (ACORFs), which represents how the ratio of IGA to LE ownership/usage changes between genders and marital status. Specifically, the left‐hand side of the models above can be rewritten in terms of a “Category Odds Ratio”:

$$\mathrm{logit}\left(\text{Pr}\left[{\mathrm{IGA}}_{i}=1\right]\right)-\mathrm{logit}\left(\text{Pr}\left[{\mathrm{LE}}_{i}=1\right]\right)=\log\frac{\mathrm{odds}[{\mathrm{IGA}}_{i}=1]}{\mathrm{odds}[{\mathrm{LE}}_{i}=1]}$$

Thus, we examine how this log ratio of IGA ownership/usage odds and LE ownership/usage odds (i.e., the log “Category Odds Ratio”) is affected by gender and marital status. In this framework, the model coefficients are no longer traditional odds ratios between two subgroups in the data but are instead measures of how this “Category Odds Ratio” differs between two subgroups. For example, a value of $\beta_{1}=2$ (i.e., ACORF = 2) would indicate that the “Category Odds Ratio” is twice as large for females than for males, controlling for the indicated factors. The exact mathematical interpretation of this coefficient is further explained in the context of the results in the next section.

After estimating the coefficients for each model specified above, we test for statistical significance using a two‐sided Wald test, chosen for its simplicity and interpretability. We perform one test at the 5% significance level per individual coefficient of interest (those attached to gender or marital status) against the null of zero effect. In addition, we also construct 95% confidence intervals for all coefficients using the same Wald method. Additional explanation and interpretation of estimated coefficients and confidence intervals is provided in the context of the results section.

We used python 3.10.9 and statsmodels 0.14.0 for all data analysis. In addition, several assumptions are required to ensure unbiased estimates of model parameters and properly compute statistical significance of these estimates. We discuss the validity of these assumptions and potential impacts on our results in the limitations section.

### 3.7 Ethics Approval

Ethics approval for the project was obtained from University of Michigan (#HUM00231049), Bishop Stuart University (BSU‐REC‐2023‐53), and the Uganda's National Council for Science and Technology (#SS1771ES). Informed consent was obtained from all participants before their inclusion in the study. Participants were fully informed of the purpose, right to withdraw, and confidentiality of their data. The study adhered to the CONSORT guidelines for transparency ensuring the methodology and results were clearly reported.

## 4 Results

The demographic characteristics of the study participants are presented in Table 1. Although we aimed to recruit 35 participants from each marital status, there was a higher turnout of married women and fewer turnout of single women and men. The total analyzed sample consisted of 100 women (44 married, 36 separated/widowed, 20 single) and 32 men. Average age for women were 42.2 years old and men were 37 years old. A significant proportion of participants belonged to Bukwali Trust Group, the largest VSLA among the five included in the study, with one‐third of women and over half of men affiliated with the group. On average, household size was approximately six people for women and five people for men and married women had the largest household size across all marital statuses. All married women indicated that their spouses were the head of household. In contrast, 88% of men stated they were head of household, while the majority of the widowed (83%) and single (70%) women reported themselves as head of households. In terms of religious affiliation, approximately 59% of women and 44% men were Catholics. In terms of education, single women had higher levels (35% secondary, 35% tertiary) followed by married women (45% primary and 32% secondary). More than half of men (56%) had secondary education. Regarding reproductive history, almost all women had been previously pregnant (91%), except single women, where 40% had never been pregnant. The most common occupation for both men and women were farming.

**Table 1:** Demographic characteristics of study participants.

	All women	All men	Married
Total counts	100	32	44
Age			
Mean (SD)	42.2 (14.8)	37.0 (11.5)	38.4 (12.1)
VSLA^1^ membership			
Divine Mercy Group	12 (12%)	5 (16%)	2 (05%)
Rwengaju	17 (17%)	–	7 (16%)
Kyamukube Smart Weavers Group	22 (22%)	3 (09%)	14 (32%)
Nyakashanja Turibamwe Group	16 (16%)	5 (16%)	8 (18%)
Bukwali Trust Group	33 (33%)	17 (53%)	13 (30%)
Missing	–	2 (06%)	–
Household size			
Mean (SD)	5.9 (2.5)	5.2 (2.7)	6.5 (2.4)
Household head			
Me	44 (44%)	28 (88%)	–
My Spouse	45 (45%)	1 (03%)	44 (100%)
My/Spouse's Parents/Grandparents	9 (09%)	3 (09%)	–
Missing	2 (02%)	–	–
Religion			
Roman Catholic	59 (59%)	14 (44%)	27 (61%)
Anglican/Protestant	27 (27%)	6 (19%)	12 (27%)
Pentecostal	9 (09%)	5 (16%)	3 (07%)
Muslim	4 (04%)	6 (19%)	2 (05%)
Other	–	1 (03%)	–
Missing	1 (01%)	–	–
Education			
None	11 (11%)	3 (09%)	3 (07%)
Primary	37 (37%)	5 (16%)	20 (45%)
Secondary	34 (34%)	18 (56%)	14 (32%)
Tertiary or University	16 (16%)	6 (19%)	6 (14%)
Missing	2 (02%)	–	1 (02%)
Previous pregnancy			
No	8 (08%)	5 (16%)	–
Yes	91 (91%)	23 (72%)	44 (100%)
Missing	1 (01%)	4 (12%)	–
Jobs^2^			
Farming	22 (22%)	7 (22%)	9 (20%)
Raising animals	5 (05%)	2 (06%)	3 (07%)
Roadside trading	1 (01%)	–	1 (02%)
Casual work for other people	2 (02%)	–	1 (02%)
Teacher	3 (03%)	–	–
Healthcare worker	9 (09%)	–	6 (14%)
Business/runs a shop	7 (07%)	4 (12%)	4 (09%)
Boda boda cyclist	–	6 (19%)	–
Hawking	7 (07%)	1 (03%)	5 (11%)
Hand craft	9 (09%)	3 (09%)	3 (07%)
I currently do not have a job	1 (01%)	–	1 (02%)
Other	3 (03%)	2 (06%)	–
Missing	31 (31%)	7 (22%)	11 (25%)

*Note:* Percentages in parenthesis are column percentages for each question. Zeros are indicated by a dash.

^1^ Village Savings\and Loan Association.

^2^ Select all that apply question, percentages may not sum to 100.

**Figure 1:**
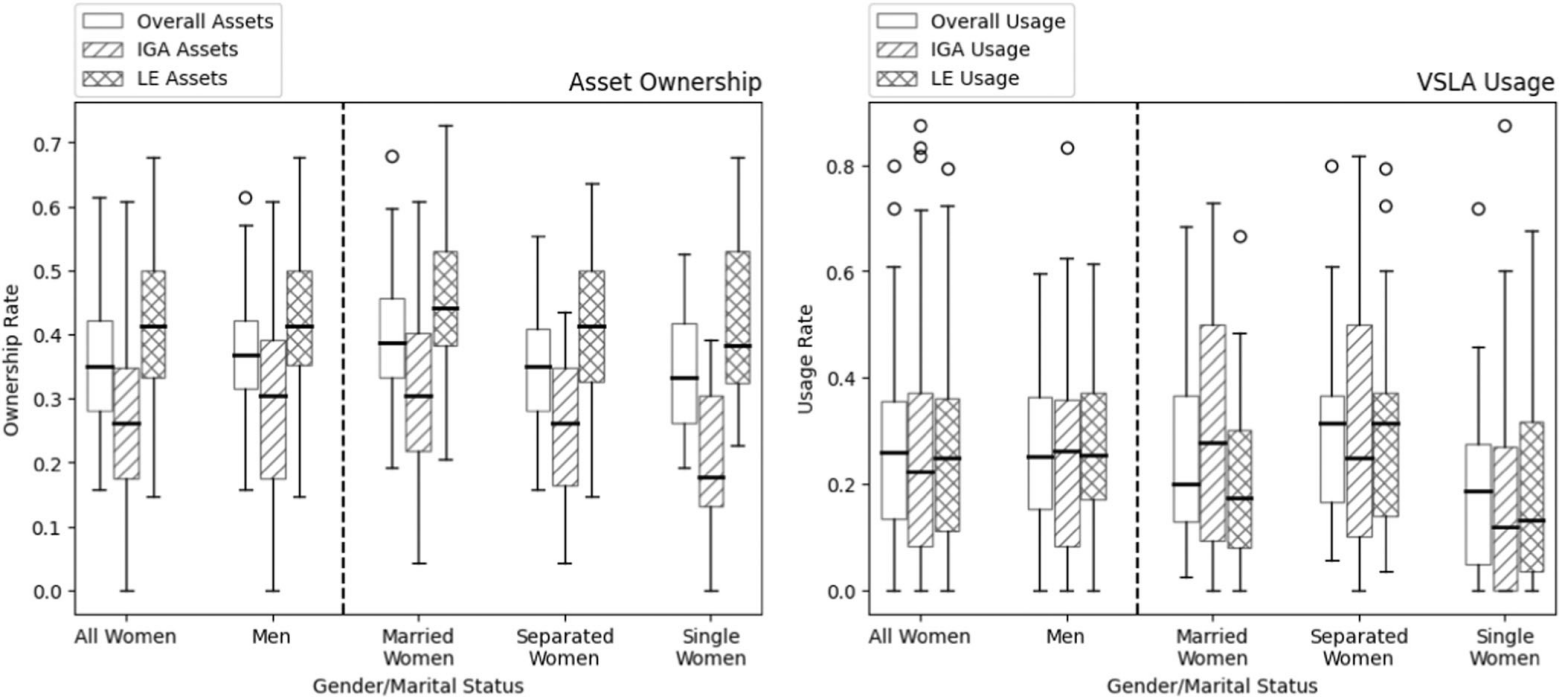
Distribution of the asset ownership and VSLA usage across gender and marital status. *Ownership rates are not adjusted for any particiapnt level factors.

Figure 1 illustrates the distribution of asset ownership and VSLA usage based on gender and marital status. In terms of asset ownership (left figure), men exhibit slightly higher ownership of overall assets, IGA assets, and LE assets compared to women. Among women, married women surpass separated/widowed women and single women in asset ownership. Single women own the fewest assets across all three types of asset variables, with most notable difference in IGA assets, where the median ownership rate is over 10% lower than that of married women. In terms of VSLA usage (right figure), women generally utilize VSLA funds more frequently than men, except for IGAs. Among women with different marital statuses, separated/widowed women are the most active users of VSLA funds followed by married women and then single women. In summary, women, on average, own fewer assets compared to men but demonstrate higher usage of VSLA funds, particularly for LEs.

Table 2 shows the estimated AORs for asset ownership and VSLA usage. For example, the AOR of 0.81 for overall assets (first column) and women versus men (first row) indicates that the odds of a woman owning any asset in the study is estimated to be 0.81 times the odds of a man owning any asset in the study. The AOR of 0.83 in the same column for single vs married women (last row) indicates that the odds of a single woman owning any asset in the study is estimated to be 0.81 times the odds of a married woman owning any asset in the study. The 95% confidence interval for the first AOR above (0.81) is given as (0.71, 0.92). If our assumptions underlying our statistical methods are valid, then the true AOR, which differs from our estimated AOR due to randomization in the study participants, falls somewhere between 0.71 and 0.92 with 95% confidence. Like the AOR estimate, the confidence interval is also affected by data randomization; 95% confidence means that, if we were to repeat this study many times with new participants and construct more confidence intervals the same way, we expect 95% of these confidence intervals to contain the true AOR.

**Table 2:** Adjusted odds ratio for asset owned and VSLA^1^ usage.

AOR	Overall assets	IGA^2^ assets	LE^3^ assets	Overall usage	IGA usage	LE usage
Women versus men^4^	0.81*	0.60*	0.96	1.40*	1	1.61*
	(0.71, 0.92)	(0.48, 0.74)	(0.81, 1.13)	(1.16, 1.70)	(0.69, 1.46)	(1.29, 2.01)
Separated versus married^5^	0.92	0.92	0.91	1.11	1.11	1.1
	(0.80, 1.06)	(0.73, 1.17)	(0.76, 1.09)	(0.93, 1.34)	(0.76, 1.64)	(0.89, 1.37)
Single versus married^6^	0.83	0.68*	0.9	0.84	1.07	0.79
	(0.70, 0.98)	(0.50, 0.92)	(0.73, 1.12)	(0.65, 1.08)	(0.63, 1.82)	(0.60, 1.05)

*Note:* 95% confidence intervals shown in parentheses. All adjusted for age, VSLA group membership, household size, religion, and education level.

^1^ Village savings and loan association.

^2^ Income generating activities.

^3^ Life events.

^4^ Adjusted odds ratios in this row are odds for all women divided by odds for all men.

^5^ Adjusted odds ratios in this row are odds for separated women divided by odds for married women.

^6^ Adjusted odds ratios in this row are odds for single women divided by odds for married women.

* *p* < 0.05.

Furthermore, Table 2 indicates that women own significantly fewer overall assets and IGA assets compared to men. However, women's overall VSLA usage and LE usage surpass those of men significantly. Specifically, for overall assets, the odds of women owning them are 19% (AOR: 0.81; 95% CI: 0.71–0.92) lower than the odds of men. Additionally, the odds of women owning IGA assets are 40% lower (AOR: 0.60; 95% CI: 0.48–0.74) compared to men. In contrast, the odds of women using VSLA funds are 40% higher (AOR: 1.40; 95% CI: 1.16–1.70) than men and 61% higher (ARO: 1.61; 95% CI: 1.29–2.01) for LEs. Among the marital statuses, married women generally own more assets than separated/widowed women and single women. However, only the difference in assets between single and married women reached statistical significance at the 5% level, with single women owning fewer overall (AOR: 0.83; 95% CI: 0.70–0.98) and IGA assets (AOR: 0.68 95% CI: 0.50‐0.92). No statistically significant difference in VSLA fund usage was observed based on women's marital status.

Lastly, Table 3 highlights the difference in the ratio of IGA and LE asset ownership and VSLA usage based on gender and marital status. The ACORF estimates attempt to quantify this difference. For example, the estimated ACORF for asset ownership (first columns) between women and men (first row) is 0.62. To interpret this estimate, we start with the “Category Odds Ratio”, which is the odds of owning an IGA asset divided by the odds of owning an LE asset. A higher “Category Odds Ratio” indicates a stronger preference towards owning IGA assets versus LE assets. Finally, the estimated ACORF of 0.62 indicates that the “Category Odds Ratio” for women is 0.62 times that for men, indicating that women have a stronger preference toward owning LE assets than men. For marital status, taking married women as the baseline, the results display that while single women's asset ownership tends more toward LE, their VSLA usage leans toward IGA. Comparisons between married and separated women are not statistically significant among results. VSLA participating women own fewer assets compared to VSLA participating men but utilize VSLA funds more than men, particularly for LEs. Among women with different marital statuses, single women own fewer assets compared to married women, with their asset ownerships leaning toward LEs. However, their VSLA usage leans toward IGAs compared to married women.

**Table 3:** Balance of IGA^1^ and LE^2^ ownership and VSLA^3^ usage.

Adjusted category odds ratio factor (ACORF)^4^	Asset ownership	VSLA^3^ usage
Women versus Men^5^	0.62*	0.59*
	(0.49, 0.77)	(0.39, 0.89)
Separated versus married^6^	1	1.12
	(0.79, 1.27)	(0.71, 1.74)
Single versus married^7^	0.73*	1.84*
	(0.53, 0.99)	(1.00, 3.40)

*Note:* 95% confidence intervals shown in parentheses.

^1^ Income generating activities.

^2^ Life events.

^3^ Village savings and loan association.

^4^ Adjusted for age, VSLA group membership, household size, religion, and education level.

^5^ ACORFs in this row are (all women IGA odds/all women LE odds)/(all men IGA odds/all men LE odds).

^6^ ACORFs in this row are (separated women IGA odds/separated women LE odds)/(married women IGA odds/married women LE odds).

^7^ ACORFs in this row are (single women IGA odds/single women LE odds)/(married women IGA odds/married women LE odds).

* *p* < 0.05.

## 5 Discussion

To the best of our understanding, this study is one of the first research studies that examined gender and marital status in its relation to VSLA participants' asset ownership and fund usage. Results found that a VSLA participants' gender and marital status significantly influence their asset ownership and VSLA usage. Analyses found that VSLA participating women owned significantly fewer assets compared to VSLA participating men, particularly for IGA assets. The prevalence of men owning more IGA assets aligns with sociocultural norms designating men as primary breadwinners [26]. However, our sample included widowed/separated women and single women, who, in many cases serve as sole breadwinner without a husband. The finding that these women have limited IGA assets that can bring in sustained income is concerning. Additionally, our study indicates that women access VSLA funds more frequently than men, but notably, this is only observed in relation to LEs usage and not IGA usage. Other studies conducted in Africa suggest that VSLAs empower women financially by enabling investing in IGAs [27, 28]. However, our study found that when women's utilization of VSLA funds for IGA related expenses is compared to that of men, significant disparity emerges, and the positive impact other studies found may not be as significant as we wish. Women not only own significantly fewer IGA assets but also utilize VSLA funds less for IGA expenses than their male counterpart.

Study results illustrate that women predominantly use VSLA funds to address various LEs, indicating that VSLA serves as informal social protection for women. This aligns with existing literature highlighting the role of SCGs in providing social protection for women by smoothing incomes and offering emergency funds for funeral assistance, food, illness, and childbirth, while additional research in Northern Ghana demonstrated that VSLAs not only enhance financial resilience but also serve as adaptive strategies to mitigate food insecurity during environmental and economic shocks [3, 28, 29]. The established tendency of women to allocate finances towards children's education, healthcare, food, and housing is a key reason why SCGs often primarily target women over men [30]. While accessing VSLA funds for social protection is crucial, there is a need for women to channel these resources into activities that can yield sustainable income. However, such investment needs to be accompanied by intentional planning and enhancement of financial literacy since literature also suggest that when women invest in small scale IGAs, the economic benefit from such investment is not large [31]. Given our intentional inclusion of widowed/separated women and single women, who serve as the heads of households without male contribution to household income, it is imperative to critically examine the reasons, barriers, and facilitators influencing women's ability to invest in IGAs.

Among women with different marital statuses, it was found that single women own significantly fewer assets, especially related to IGAs. Despite facing higher levels of economic disadvantage compared to married women, single women exhibit a notable trend of utilizing VSLA funds extensively for IGA than for LEs compared to married women. This unique finding suggests that while single women face greater economic challenges, they actively invest in and develop IGAs. Several factors may contribute to this trend such as potential younger age, higher education level, and smaller household size of single women. These factors align with a study conducted in Kenya, which found that women with dependents tend to allocate more money on food and school fees, while those without dependents direct funds towards investment and clearing health bills [5]. Although widowed/separated women and single women in our study had similarly smaller household sizes compared to married women and men, the combination of the other demographic variables may have contributed to the observed significant difference. Further exploration of these factors could provide valuable insights into the unique economic dynamics of single women within VSLA programs.

Our study highlights the significant influence of gender and marital status on asset ownership and VSLA fund usage. Notably, there is a scarcity of research that compares women's financial empowerment through VSLA to that of VSLA participating men. Intersectionality theory [18] helps explain how multiple, overlapping social identities–such as gender, marital status, and economic status–shape women's experiences within VSLA. The complexity of women's marital status, with its sociocultural and economic nuances, is often overlooked in studies examining its impact on VSLA participation, financial gains, and expenditures. In many African contexts, marriage is a critical determinant of women's access to social and economic rights, and the termination of marriage, through divorce or widowhood, often result in significant loss, leaving women with nothing [32]. In Uganda, where 14% of women ages 15–49 are widowed/separated, and 26% never married [22], the impact of widowhood or separation is particularly pronounced, as some women face the loss of their home, property, and social exclusion [32].

Social Capital Theory [19, 20] further contextualizes these disparities by highlighting how VSLAs serve as both financial and social support networks, but access to these benefits is not evenly distributed. Single women or women living in rented houses are often considered high risk participants, due to their potentially transitory status, which can create barriers to obtaining loans from the group [29]. In contrast, being married along with having children gave the perception that the prospective member would not flee with the group's money [29]. Consequently, unmarried single women may face exclusion and discrimination within VSLA groups [29]. While our study included widowed/separated women and single women, potential instances of discrimination and limited access to VSLA funds may not be fully captured.

Beyond marital status, economic status further intersects with gendered financial barriers, as some studies suggest that being perceived as “too poor” can also lead to exclusion from VSLAs and the broader social networks that provide financial protections [33, 34]. These findings underscore the need for future research to examine the implications of low asset ownership among women—particularly single women—through an intersectional lens. Additionally, VSLA interventions should proactively address structural inequalities by developing policies that promote equitable access to financial and social resources.

This highlights the pressing need for further research to fully capture the transformative impact of VSLAs**,** especially in comparison to men's participation and financial outcomes. Future studies should explore how gender and marital status shape various dimensions of VSLA engagement, including participation, experiences, access, and fund utilization. Moreover, research should investigate the differences between mixed‐gender and single‐status VSLA groups to assess how social dynamics influence financial inclusion and empowerment. Tailoring VSLA models to ensure universal financial inclusion—particularly for single, widowed, and economically disadvantaged women—remains a critical area for intervention. Finally, while financial inclusion efforts have largely focused on empowering women, meaningful engagement with men is equally essential. Promoting the financial inclusion of marginalized men within VSLAs can foster an environment where both genders actively support and empower one another, creating a more sustainable and inclusive approach to economic resilience and growth.

### 5.1 Limitations

This study has several limitations. Self‐reported data collected midway through the VSLA cycle is susceptible to social desirability and recall biases. However, since the research team did not implement the VSLA program, participants may have felt more comfortable providing honest responses. The sample size, comprising 100 women and 32 men from Fort Portal, Uganda, limits generalizability. Categorizing marital status into three groups oversimplifies its complex dynamics in diverse cultural contexts. Logistic regression assumes linear relationships for numerical confounders, like age, which may not reflect reality. Additionally, fungibility issues arise since we could not distinguish assets already owned prior to joining the current VSLA cycle purchased specifically with VSLA funds, potentially introducing bias. Lastly, interdependence among VSLA members' fund access might narrow confidence intervals. While acknowledging the limitations of the study, our study findings highlight the important nuances of gender and marital status in the context of VSLA by comparing the assets and VSLA usage among men, women, within the same VSLAs. Our study provides a foundation for future studies to further investigate the barriers faced by women of different marital statuses within VSLAs, considering the multifaceted influence of societal norms, economic conditions, and discriminatory practices.

## 6 Conclusion

The study offers valuable insights into the nuanced dynamics of gender and marital status within VSLA in rural Uganda. The findings underscore the significant impact of these factors on asset ownership and VSLA fund utilization among participants. Notably, women access VSLA funds more frequently compared to men but primarily for LEs rather than IGAs. Furthermore, we found that single women face distinct challenges in asset ownership yet exhibit a proactive approach in utilizing VSLA funds for IGAs and LEs. The study highlights the broader sociocultural and economic implications of marital status on women's financial agency, emphasizing the need for a more nuanced understanding of the diverse experiences of widowed, separated, and single women in VSLAs. Despite the potential of VSLAs to empower women economically, our research suggests that certain sociocultural norms and discriminatory practices may impede the full realization of financial inclusion, especially for single women.

To foster universal financial inclusion, future studies should explore strategies to tailor existing VSLA models, ensuring they address the specific needs of diverse participants. Additionally, the study underscores the importance of engaging men meaningfully in financial inclusion initiatives, fostering an environment of mutual support and empowerment. In essence, our research contributes to the growing body of knowledge of microfinance mechanisms and calls for a more equitable approach to VSLA participation, considering the intersecting factors of gender and marital status. As the world strives for inclusive financial empowerment, understanding and addressing these complexities within a low‐ and middle‐income setting, where women are most vulnerable financially, are essential for the development of interventions aimed at financial equity for all individuals.

## Author Contributions

**HaEun Lee:** conceptualization, investigation, funding acquisition, writing – original draft, methodology, data curation, supervision, writing – review and editing, visualization, validation. **Alex Shen:** writing – original draft, methodology, visualization, writing – review and editing, software, formal analysis, data curation, supervision, validation. **Alia Dada:** writing – original draft, methodology, data curation, visualization, writing – review and editing. **Isabel Gilbertson:** writing – original draft, visualization, writing – review and editing, data curation, methodology. **Cheryl A. Moyer:** conceptualization, investigation, supervision, resources, writing – review and editing, validation. **Donah Asiimire:** conceptualization, investigation, writing – original draft, methodology, writing – review and editing, data curation, supervision, resources, project administration, validation.

## Conflicts of Interest

The authors declare no conflicts of interest.

## Transparency Statement

The lead author HaEun Lee affirms that this manuscript is an honest, accurate, and transparent account of the study being reported; that no important aspects of the study have been omitted; and that any discrepancies from the study as planned (and, if relevant, registered) have been explained.

## Data Availability

The data that support the finding of this study are available from the corresponding author upon reasonable request.
